# High Adherence of Oral Streptococcus to Polylactic Acid Might Explain Implant Infections Associated with PLA Mesh Implantation

**DOI:** 10.3390/ijms24119504

**Published:** 2023-05-30

**Authors:** Sonia Sarfraz, Anni-Maria Tamminen, Junnu Leikola, Sonja Salmi, Mika Kaakinen, Timo Sorsa, Juho Suojanen, Justus Reunanen

**Affiliations:** 1 Biocenter Oulu, Cancer and Translational Medicine Research Unit, University of Oulu, 90014 Oulu, Finland; sonia.sarfraz@oulu.fi (S.S.); mika.kaakinen@oulu.fi (M.K.);; 2 Päijät-Häme Joint Authority for Health and Wellbeing, Department of Oral and Maxillofacial Surgery, Lahti Central Hospital, 15850 Lahti, Finland; annimaria.tamminen@gmail.com; 3 Cleft Palate and Craniofacial Centre, Department of Plastic Surgery, Helsinki University Hospital, 00029 Helsinki, Finland; junnu.leikola@hus.fi; 4 Oulu Centre for Cell-Matrix Research, Faculty of Biochemistry and Molecular Medicine, University of Oulu, 90220 Oulu, Finland; 5 Department of Oral and Maxillofacial Diseases, Helsinki University Hospital, 00014 Helsinki, Finland; timo.sorsa@helsinki.fi; 6 Clinicum, Faculty of Medicine, University of Helsinki, 00014 Helsinki, Finland

**Keywords:** bacterial adhesion, bioresorbable, polylactic acid, saliva, titanium

## Abstract

The aim of this study was to evaluate and compare the biofilm formation properties of common pathogens associated with implant-related infections on two different implant material types. Bacterial strains tested in this study were *Staphylococcus aureus*, *Streptococcus mutans*, *Enterococcus faecalis*, and *Escherichia coli*. Implant materials tested and compared were PLA Resorb × polymer of Poly DL-lactide (PDLLA) comprising 50% poly-L-lactic acid and 50% poly-D-lactic acid) and Ti grade 2 (tooled with a Planmeca CAD-CAM milling device). Biofilm assays were done with and without saliva treatment to evaluate the effect of saliva on bacterial adhesion and to mimic the intraoral and extraoral surgical routes of implant placement, respectively. Five specimens of each implant type were tested for each bacterial strain. Autoclaved material specimens were first treated with 1:1 saliva-PBS solution for 30 min, followed by washing of specimens and the addition of bacterial suspension. Specimens with bacterial suspension were incubated for 24 h at 37 °C for biofilm formation. After 24 h, non-adhered bacteria were removed, and specimens were washed, followed by removal and calculation of adhered bacterial biofilm. *S. aureus* and *E. faecalis* showed more attachment to Ti grade 2, whereas *S. mutans* showed higher adherence to PLA in a statistically significant manner. The salivary coating of specimens enhanced the bacterial attachment by all the bacterial strains tested. In conclusion, both implant materials showed significant levels of bacterial adhesion, but saliva treatment played a vital role in bacterial attachment, therefore, saliva contamination of the implant materials should be minimized and considered when placing implant materials inside the body.

## 1. Introduction

Polylactic acid (PLA) is a biopolymer synthesized from nontoxic renewable resources such as the fermentation of plant starch from corn, wheat, or sugarcane. PLA has been widely studied for biomedical applications in the human body based on its properties of biodegradability, biocompatibility, ability to induce bone formation, and mechanical strength [1]. Biodegradable polymers, such as PLA, degrade directly by enzymatic activity or by hydrolysis of the polymeric chain into monomers, which ultimately produce carbon dioxide and water. These degradation properties depend on several factors, including molecular structure, rate of water diffusion, and crystallinity. PLA and its composites have a range of physical, chemical, and mechanical properties that can be engineered appropriately depending on the particular application, such as pediatric surgery or orthopedics [2].

PLA has been established as a promising biomaterial due to its versatile applications in modern medicine, such as orthopedic interventions, tissue engineering, drug carriers, regenerative medicine, cancer therapy, implants, and medical equipment [3]. PLA and PLA-composite implants are used in fracture fixation in cases of orthopedics, facial, oral, and traumatology surgery in the forms of anchors, fixing joint-pins, plates, cages, meshes [4], and as a carrier for bone morphogenetic proteins and growth factors among others [5]. PLA and PGA (polyglycolic acid) are known as the first biodegradable polymers that are utilized in biomedicine. Both of these biodegradable polymers have suitable mechanical properties which enable them to be used in internal bone fixation devices. In combination, poly (lactic-co-glycolic acid) PLGA has been used in drug delivery systems as a polymeric shell. Several other biodegradable polymers are also being used in biomedical applications. These include polycaprolactone (PCL), Polybutylene succinate (PBS), chitosan, polyhydroxybutyrate (PHB), Polydioxanone (PDO), hydrogels, hyaluronic acid, polyurethanes (PU), and polyester urethanes (PEUU). PCL has been used in anti-cancer treatment drug delivery, and hyaluronic acid and chitosan are being used in ligament and cartilage repair. PU and PEUU have suitable properties and biocompatibility and are used as implant materials [6].

Another polymer, Polyhydroxyalkanoates (PHAs), has great potential to be used in medical applications due to its biodegradability. It is produced naturally by certain bacteria through the fermentation of sugars and lipids. They are being extensively used in a wide range of orthopedics and fixation devices. However, some limited aspects of PHA, such as high-cost production and bad mechanical and thermal properties, make it less suitable in comparison with PLA [7].

Over the years, titanium and other metal screws and plates have been used as internal fixtures since they provide quick and solid fixation. A study investigated titanium (Ti6Al4V) and other metals such as cobalt chromium molybdenum (CoCrMo) and stainless steel (SS 316L) and compared their properties as medical implant material in total hip implants regarding contact pressure. This study concluded the use of titanium as the suitable material for metal-on-metal application reducing contact pressure by up to 35% in contrast to the other metals tested [8]. However, metal implant materials pose several risks, including protrusion, foreign body reaction, palpation, and a need for additional surgery to remove the implant. Resorbable implant materials such as PLA and others complement these disadvantages of metal implants [9]. The role of resorbable mesh as a fixation device in craniosynostosis and fracture of craniofacial bone has been reported as a successful and secure fixation method [9,10].

Implant-related infection is a major complication in implantation surgeries and is caused by bacterial biofilm attachment on the implant surface, which renders the healing process and success of implantation. It can result in the removal of implanted devices along with antimicrobial treatment for prolonged periods [11]. The complex process of bacterial adhesion is influenced by material surface properties, environmental factors, bacterial properties, and the presence of specific proteins at the infection site [12].

Studies conducted on PLA and its copolymers, such as PGA, in surgical procedures concluded its most frequent application as a resorbable membrane in different procedures. There is limited evidence to support the influence of infection or possible bacterial contamination in the field of biodegradable implants. However, few studies mentioned infection after using resorbable PLA material [13,14]. The previous literature and research on the use of PLA in biomedical applications reported varied results. For example, some studies concluded that the use of PLA in cranioplasties, orthognathic, and maxillofacial surgeries is safe and is associated with a low rate of complications as compared to titanium. Whereas several other studies reported a high rate of infection and complications related to the use of PLA. Prior studies on PLA and limitations will be discussed in more detail in the Section 3.

The aims of this study were to assess and compare the bacterial biofilm formation of common implant-associated pathogens such as *Staphylococcus aureus*, *Streptococcus mutans*, *Enterococcus faecalis*, and *Escherichia coli* on PLA and titanium grade 2 and to test the effect of salivary contamination on later bacterial adhesion to the material. Biofilm formation experiments were carried out with and without saliva treatment to imitate both intraoral and extraoral surgical routes for implant placement.

## 2. Results

### 2.1. Biofilm Formation

In non-saliva treated specimens, biofilm formation of *S. aureus*, *E. faecalis*, and *E. coli* showed no significant difference between titanium and PLA. However, *S. mutans* adhered more strongly to PLA than titanium in the non-saliva-treated group (*p* ≤ 0.01).

In the saliva-treated group, *S. aureus* was found to be more adhered to titanium as compared to PLA (*p* ≤ 0.05), whereas *S. mutans*, *E. faecalis*, and *E. coli* did not show any significant difference in adherence between materials after saliva contamination.

When comparing non-saliva and saliva-treated specimens, saliva contamination showed a major effect on bacterial attachment by enhancing biofilm formation. For example, in *S. aureus*, saliva-treated Ti specimens showed higher attachment than non-saliva-treated Ti and PLA (*p* ≤ 0.001) and (*p* ≤ 0.01), respectively. Similarly, saliva-treated PLA showed higher biofilm formation than non-saliva-treated specimens of PLA (*p* ≤ 0.05) and Ti (*p* ≤ 0.05).

With *S. mutans*, saliva contamination also increased bacterial attachment to PLA and Ti. Saliva-treated PLA had higher bacterial adherence as compared with non-saliva-treated PLA (*p* ≤ 0.05) and Ti (*p* ≤ 0.001). The salivary coating on Ti grade 2 also showed higher attachment than PLA (*p* ≤ 0.05) and Ti (*p* ≤ 0.001) by *S. mutans*.

In the case of *E. faecalis*, saliva-treated PLA presented higher biofilm formation than non-saliva-treated PLA (*p* ≤ 0.01) and Ti (*p* ≤ 0.001). Similarly, saliva-treated Ti showed higher adherence than non-saliva-treated PLA (*p* ≤ 0.001) and Ti (*p* ≤ 0.01).

With *E. coli*, saliva contamination in PLA and Ti caused higher biofilm formation than non-saliva-treated specimens, but this difference was not statistically significant except for saliva-treated Ti, which had a significant difference (*p* ≤ 0.05) against non-saliva-treated PLA.

Overall, all bacterial strains were attached to both materials with high density, but the majority of the strains were found to be more adherent to Ti grade 2 except for *S. mutans*, which showed significantly higher adhesion to PLA than to titanium under non-salivary conditions.

**Figure 1:**
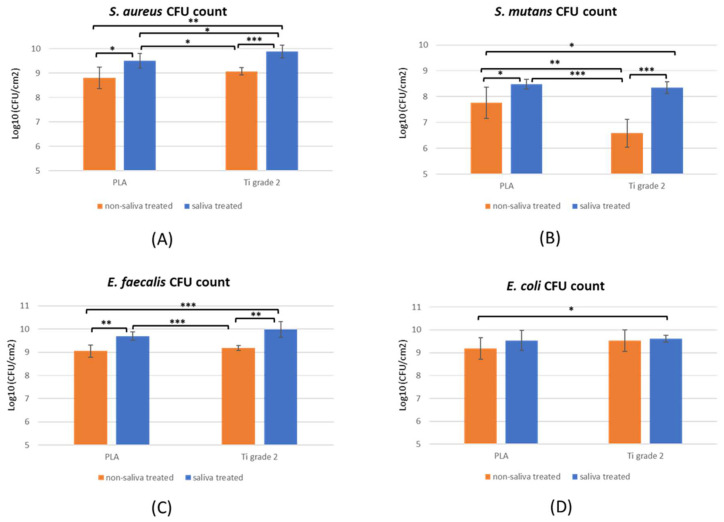
Colony forming unit (CFU) count of (**A**) *S. aureus*, (**B**) *S. mutans*, (**C**) *E. faecalis*, and (**D**) *E. coli* on PLA and Ti grade 2. Statistical difference between materials is marked with lines * *p* ≤ 0.05, ** *p* ≤ 0.01, *** *p* ≤ 0.001.

Bacterial biofilm assay results with and without saliva treatment of specimens are shown in Figure 1.

### 2.2. SEM Analysis of Biofilms

**Figure 2:**
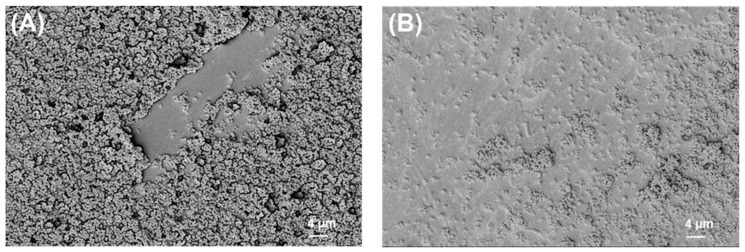
*S. aureus* biofilm on (**A**) PLA and (**B**) Ti grade 2. Magnification = 1.18 KX with scale bars = 4 μm.

**Figure 3:**
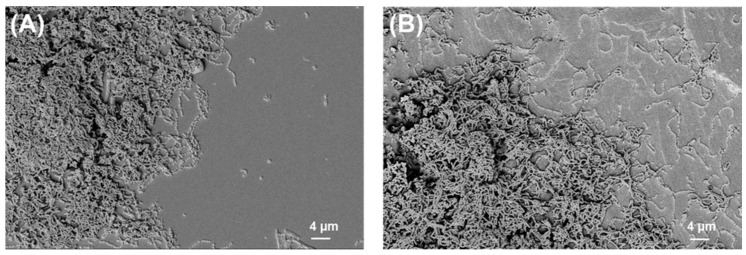
*S. mutans* biofilm on (**A**) PLA and (**B**) Ti grade 2. Magnification = 1.18 KX with scale bars = 4 μm.

**Figure 4:**
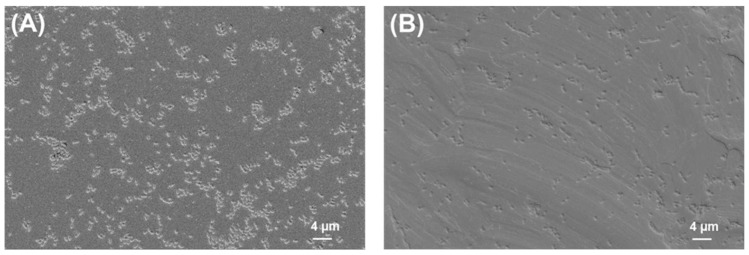
*E. faecalis* biofilm on (**A**) PLA and (**B**) Ti grade 2. Magnification = 1.18 KX with scale bars = 4 μm.

**Figure 5:**
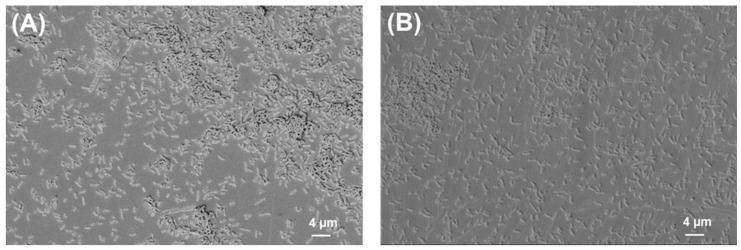
*E. coli* biofilm on (**A**) PLA and (**B**) Ti grade 2. Magnification = 1.18 KX with scale bars = 4 μm.

The SEM images of bacterial biofilms on PLA and titanium grade 2 are shown below (Figure 2, Figure 3, Figure 4 and Figure 5).

## 3. Discussion

In this study, we assessed and compared the bacterial biofilm formation of common surgical implant materials used for osteosynthesis or bone contouring in orthopedics and cranio-maxillo-facial surgery. Surgical site infection-associated pathogens biofilm formation on PLA and titanium was assessed, and we also tested the effect of salivary coating on bacterial adhesion in vitro. This study finds that the biofilm formation of *S. mutans* favors PLA over titanium, whereas *S. aureus* binds more strongly to titanium. Another major result was that salivary coating increased biofilm formation on both implant materials tested by all four bacterial strains in a statistically significant manner, which is in accordance with our previous studies [15,16]. Another study has also reported high bacterial adherence to implant material with saliva contamination [17], which might be because the presence of salivary pellicles on the implant surface reduces the chemical charge and make it more hydrophilic to favor the attachment of bacteria. Bacterial attachment to pellicles is mediated by several major protein adhesins produced by bacterial species. The acquired salivary pellicle contains salivary proteins along with the proteins and other molecules from the mucosa, food, and gingival fluid in the oral cavity. The main components of pellicles which act as ligands to bacterial adhesins are collagen, fibrinogen, glycolipids, and carbohydrates [18].

Titanium fixation is the gold standard in oral maxillofacial surgery [19], and it takes place in the facial area both inside and outside the oral cavity. Fixation materials in maxillofacial surgery are used in fracture fixation, orthognathic surgery, and different kinds of cancer-related reconstruction. In orthognathic surgery, different osteotomies are performed to change the relation of jaws to achieve a more functional biting for the patient. Oral maxillofacial surgery fractures and orthognathic surgery osteotomies are usually fixed with titanium plates and screws. However, there are some problems with titanium as a fixation material, such as palpability, the need to remove the material afterward, temperature sensitivity, and growth restriction [9], among others. Absorbable biomaterials such as PLA are developed to offer a solution to these problems.

In orthognathic surgery, PLA-based bioabsorbable fixation materials have no difference from titanium in terms of infection as a complication [20,21,22,23,24]. Contrary to our clinical experience in oral maxillofacial fracture surgery, no major difference between the two materials was pointed out [25,26,27]. However, the removal of titanium plates was found to be more frequent than the removal of PLA plates afterward [19,25,26].

In a meta-analysis, Yang et al. [24] noted that PLA materials used in maxillofacial surgery had a slightly higher rate of complications when it comes to mobility and foreign body reactions compared to titanium. In the analysis, infection and wound dehiscence were also detected more in the bioabsorbable group compared to the titanium group. However, this finding was not statistically significant. In contrast, a recent broad review article conducted on the subject suggests that the clinical use of PLA in maxillofacial surgery is safe and comparable to titanium in many cases [19].

In craniofacial surgery, PLA materials have been in clinical use for calvarial osteosynthesis for decades. This is especially beneficial in the pediatric population that presents the majority of cranial surgery patients [28]. Kalmar et al. also reported that the PLA resorb material has minimal or no influence on cranial growth [29]. Unlike in maxillofacial surgery, cranial PLA plates very seldom become contaminated by the surrounding flora and are, therefore, less likely to have a bacterial infection. However, the blood flow of the surrounding skin might be compromised if large cranial expansions are performed, causing tension in the skin. There is normally very little functional load or stress on the PLA plates and, therefore, normally relatively thin plates may be used. This reduced PLA material causes a smaller inflammation due to resorption and, therefore, has a lower risk of being exposed. Serlo et al. [30] have indicated in their study the beneficial effect of placing the PLA plates on the interior surface of the calvarial, but its possible effect on the underlying dura is still unclear.

While there are already studies about coating or loading PLA with different antibacterial substances, such as prodigiosin vitamin-E and barium titanate, among others, to reduce the bacterial adhesion to the surface of the material [31,32,33], future research should be conducted in the field of making PLA more resistant to bacterial biofilm formation.

Previous clinical experience of using PLA osteosynthesis in maxillo-facial trauma and especially open facial fractures with traumatic wounds are associated with a rather high infection rate and compromised wound closure. On the contrary, wound infections in PLA material are rare in cranioplasties with sterile surgical wounds and primary closure. Interestingly, no evident clinical data supports our clinical findings. Whether it is only our speculation or possibly a result of negative publication bias remains to be investigated.

There are certain limitations in the present in vitro study. It is important to understand that biofilm formation in in vitro conditions lacks certain factors that are found in in vivo conditions, such as the presence of host immune response and the presence of multiple microbial species in polymicrobial biofilms. The bacterial adherence to the material sets some limitations for the use as it might increase the risk of infection. Considering our results, special attention should be given when treating immunocompromised patients with PLA-based bioabsorbable materials. PLA might still not be the material for these patients. When using PLA in surgery as a fixation material, extra care should also be taken for aseptic and hygienic procedures. Most importantly, open salivary contamination should be minimized. More studies of PLA developments with antibacterial substances are required to get these hybrid materials in action.

## 4. Materials and Methods

### 4.1. PLA and Titanium Implant Preparation

The implant types studied were PLA and titanium grade 2. PLA implant material used in this study was Resorb x KLS martin, Resorb x, REF 52-303-51-04, 78532 Tuttlingen/Germany). Resorb x is a polymer of poly DL-lactide (PDLLA) comprising 50% poly-L-lactic acid and 50% poly-D-lactic acid. The titanium implant used in this study was from CAD-CAM technical provider Planmeca Ltd. (Helsinki, Finland). These implants were manufactured as 20 mm discs, sterilized, and destained in a similar way as they are normally provided for the needs of the patients. Five specimens/implant types were tested in bacterial adhesion experiments.

### 4.2. Bacterial Suspension Preparation

Four bacterial strains were tested for their adhesion properties. *S. aureus* (DSM 29134), *S. mutans* (DSM 20523), and *E. faecalis* (DSM 20380) were bought from Leibniz Institute DSMZ-German Collection of Microorganisms and Cell Culture GmbH, and *E. coli* was isolated from a human fecal sample. *S. aureus*, *S. mutans*, and *E. faecalis* were cultured in Trypticase Soy Yeast extract medium, and for *E. coli*, Lysogeny broth (LB) was used.

Bacterial adhesion experiments of all strains were done following the same protocol. Bacterial cultures were grown overnight and centrifuged at 8000× *g* for 10 min to form a bacterial pellet, resuspended in 1 × PBS (phosphate buffer saline), and centrifuged again. The washed bacterial pellet was then diluted by using respective growth media to OD600 = 0.25.

### 4.3. Saliva Contamination 

Biofilm formation on implants was carried out with and without saliva contamination of the implants. Saliva treatment of the implants was done prior to the addition of bacterial culture for biofilm formation. Sterile saliva was collected from healthy volunteers (Ethics Committee of the Northern Ostrobothnia Hospital District, EETTMK 11/2019) using a paraffin wax stimulation. Collected saliva was pooled and filtered using a 0.45 µm filter (#167-0045 Nalgene™ Rapid-Flow™ Sterile Single Use Vacuum Filter Units, Nalgene^®^ 295-4545, Edo, de México, Mexico) followed by storage at −80 °C. Before the procedure, filtered saliva was diluted 1:1 in 1 × PBS. The pH of saliva was measured before the addition of buffer (7.6) and after the addition of buffer (7.5), which was within the range of normal pH of saliva (6.2–7.6). Sterile PLA and autoclaved titanium specimens were first treated with saliva by immersing into the saliva-PBS solution for 30 min, followed by washing with 1 × PBS.

### 4.4. Biofilm Formation and Enumeration of Adhered Bacteria

Implant material specimens were first treated with saliva-PBS solution for saliva contamination. Specimens with and without saliva treatments were transferred to Petri plates. Bacterial culture was added to the plates and sealed with parafilm to incubate at 37 °C, and biofilm was allowed to form for 24 h.

To calculate the adhered bacteria after 24 h, bacterial suspension was carefully removed, and specimens were washed 3 times with 1 × PBS to remove non-adhered bacterial cells. Washed specimens were transferred to 6-well culture plates containing 1 specimen/well. To remove the attached biofilm to the specimens, 1 mL of 1 × PBS was added to each well and scrapped with dental brush sticks (GUM^®^ SOFT-PICKS^®^ ORIGINAL, SUNSTAR Deutschland GmbH, Schwarzwald, Germany). Then, 1 × PBS containing detached biofilm along with dental brush tips were collected into Eppendorf tubes. Tubes were then vortexed vigorously to remove bacterial cells from the tips. Adhered bacterial cells were enumerated by making serial dilutions and Colony Forming Unit (CFU) counting using respective growth media, i.e., trypticase soy yeast extract medium agar and LB agar medium. CFU count was calculated after incubation of plates for 48 h at 37 °C.

**Figure 6:**
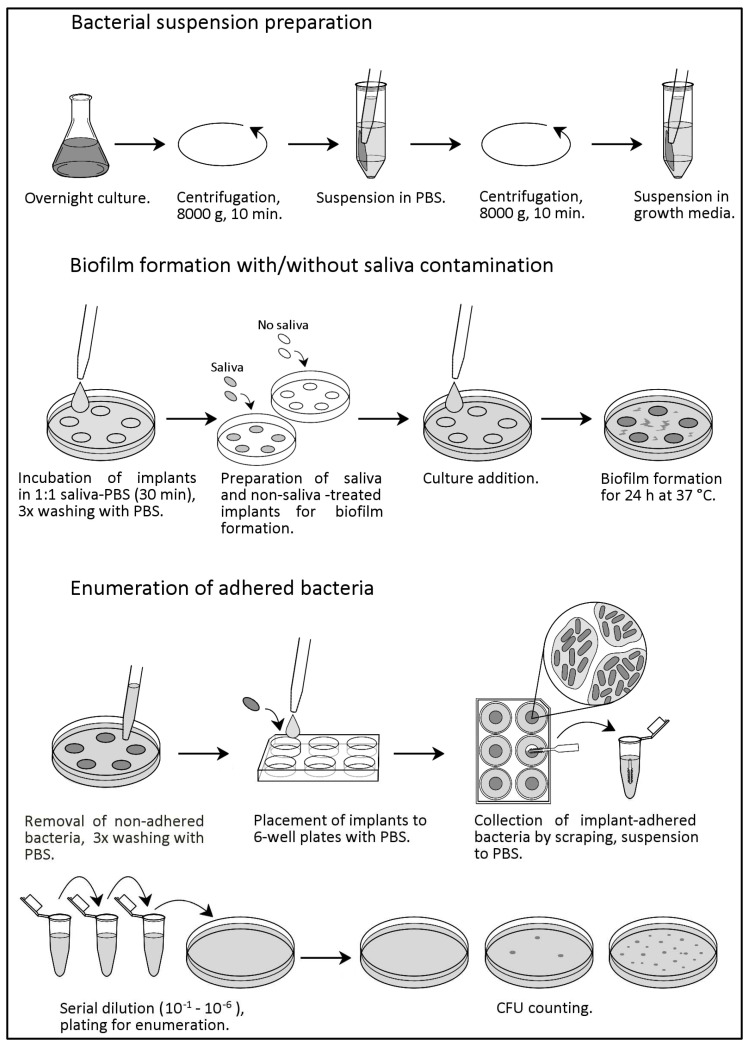
Experimental workflow of the sample preparation and biofilm assay.

The schematic workflow of this study is illustrated in Figure 6.

### 4.5. Preparation of Samples for Scanning Electron Microscopy (SEM)

Bacterial biofilm was allowed to form on the specimens under the growth conditions specified above for 24 h, followed by the removal of non-adherent bacteria by washing with 1 × PBS. The specimens with attached biofilms were prepared for SEM by fixing with 1% glutaraldehyde and 4% paraformaldehyde in 0.1 M phosphate buffer, air dried, and then sputter coated with a 5 nm platinum layer. Imaging was done with Sigma HD VP FE-SEM by using an In-Lens detector.

## 5. Conclusions

Based on the present study, it can be concluded that the implant material type has rather little effect on bacterial adhesion, but the salivary coating on the implant surface significantly increased bacterial attachment. Hence, implantation using PLA, especially in immunosuppressed patients, should be done with special attention, and saliva contamination should be minimized. Further research needs to be carried out for the development of PLA, such as the antimicrobial coating on PLA to minimize implant infections and failure.

## Data Availability

Not applicable.
